# Information and Communication Technologies, a Promising Way to Support Pharmacotherapy for the Behavioral and Psychological Symptoms of Dementia

**DOI:** 10.3389/fphar.2019.01122

**Published:** 2019-09-30

**Authors:** Antoine Piau, Pierre Rumeau, Fati Nourhashemi, Maria Soto Martin

**Affiliations:** ^1^Gérontopôle, CHU Toulouse, Toulouse, France; ^2^Oregon Center for Aging & Technology (ORCATECH), Oregon Health & Science University, Portland, OR, United States; ^3^UMR 1027, INSERM, Toulouse, France

**Keywords:** remote follow-up, monitoring, digital biomarkers, behavioral and psychological symptoms of dementia, pharmacology, clinical trials, sensors, technology

## Abstract

Health care systems face an expansion in the number of older individuals with a high prevalence of neurodegenerative diseases and related behavioral and psychological symptoms of dementia (BPSDs). Health care providers are expected to develop innovative solutions to manage and follow up patients over time in the community. To date, we are unable to continuously and accurately monitor the nature, frequency, severity, impact, progression, and response to treatment of BPSDs after the initial assessment. Technology could address this need and provide more sensitive, less biased, and more ecologically valid measures. This could provide an opportunity to reevaluate therapeutic strategies more quickly and, in some cases, to treat earlier, when symptoms are still amenable to therapeutic solutions or even prevention. Several studies confirm the relationship between sensor-based data and cognition, mood, and behavior. Most scientific work on mental health and technologies supports digital biomarkers, not so much as diagnostic tools but rather as monitoring tools, an area where unmet needs are significant. In addition to the implications for clinical care, these real-time measurements could lead to the discovery of new early biomarkers in mental health. Many also consider digital biomarkers as a way to better understand disease processes and that they may contribute to more effective pharmaceutical research by (i) targeting the earliest stage, (ii) reducing sample size required, (iii) providing more objective measures of behaviors, (iv) allowing better monitoring of noncompliance, (v) and providing a better understanding of failures. Finally, communication technologies provide us with the opportunity to support and renew our clinical and research practices.

## Context: Behavioral and Psychological Symptom Treatment in Clinical Care

Health care systems are facing a rapid increase in the number of older people with a high prevalence of neurodegenerative diseases and related behavioral and psychological symptoms of dementia (BPSDs) (Okura and Langa, 2011; Peters et al., 2015; World Alzheimer Report 2018, 2018). Behavioral and psychological symptoms of dementia are frequent, associated with faster disease progression and increased caregiver burden and health care costs (Schneider et al., 2006; Costa et al., 2018; Kales et al., 2019). Health care providers are expected to develop their ability to follow up patients at home after treatment is initiated and to adjust their treatments accordingly over time.

Apart from direct but episodic observation of behavior and mental states by the prescribing physician, the assessment of BPSDs is mainly based on reported information (by a professional or family caregiver). In addition to the information bias (Lyketsos, 2015), the episodic and evolving nature of BPSDs makes it difficult to reliably quantify their frequency and severity over time. Moreover, this assessment is often conducted in settings (health care settings) that can influence patients’ behavior and thus distort conclusions (Krolak-Salmon et al., 2016). All these care-related issues are shared by pharmaceutical research. Beyond dropout rates and loss of follow-up, monitoring compliance with treatment plans and managing potential adverse drug reactions are major concerns. Researchers recommend and encourage the search for new monitoring outcomes, such as those based on new technologies (Soto et al., 2014; Soto et al., 2015; Sano et al., 2018). In addition, it is becoming increasingly difficult to ignore the importance of real-world data (McDonald et al., 2016), which lies between controlled clinical trials with highly selected participants and clinical care, for clinical research. Real-world data include all data not collected in the context of a randomized controlled trial (e.g., postmarketing drug safety), and technological advances could be a way to remotely collect and analyze this information.

Researchers and clinicians face the same difficulties in accurately monitoring symptom progression and response to treatment over time in real life setting (in the patient’s own environment). They could benefit from complementary solutions allowing the remote collection of continuously updated and objectively measured data. Information and communication technologies (ICTs) could play a crucial role.

## Current Technological Advances in the Remote Assessment and Monitoring of BPSDs

Beyond the potential organizational benefits (frequent and massive collection at lower cost), remote evaluation of personal data could provide more sensitive and ecologically valid measures (Wild et al., 2008). It is possible to passively collect data on a patient’s behavior (e.g., sleep or motor activity) in his/her own environment or to collect information longitudinally *via* his/her caregiver through, for example, semiautomated questionnaires on a smartphone without the need to move the patient. This could limit the potential negative impact of the environment on the measure (Krolak-Salmon et al., 2016). Remote data collection could complement traditional care, providing potentially less biased and more in-depth information and nontraumatic care. Although some studies suggest the validity of computer-based tests (Wild et al., 2008; Rentz et al., 2013; Myers et al., 2016) and telemedicine (Ramos-Ríos et al., 2012; Martin-Khan et al., 2012) for cognitive evaluation, most initiatives do not exploit the potential of remote assessment at home and home-based studies generally focused on caregiver support (e. g., training and online support platform) (Boots et al., 2014).

However, research teams are increasingly interested in the possibilities of remote evaluation and monitoring of BPSDs (Mallo et al., 2018; Gibson and Gander, 2019; Gaugler et al., 2019; Nesbitt et al., 2019). The Mild Behavioral Disorder Checklist, administered remotely by telephone, is sensitive to the detection of mild behavioral disorders in people with Mild Cognitive Impairment (MCI) (Mallo et al., 2018) but is not designed for home monitoring. Recently, a randomized controlled pilot trial evaluated the effect of using WeCareAdvisor, an innovative online tool designed to enable caregivers to monitor and manage BPSDs in the home (Kales et al., 2018), with encouraging results. Megges et al. (2018) evaluated wearable global positioning system (GPS) devices for persons with dementia and their caregivers without being able to draw any conclusions in terms of effectiveness. Several other ongoing studies involve remote monitoring of BDSP at home using ICTs, suggesting that the field will evolve rapidly in this direction. In the ongoing FamTechCare study (Williams et al., 2018), caregivers create video recordings of difficult care situations, and a team of experts reviews the videos and proposes interventions. Wallack et al. (2018) are evaluating the value of the expertise provided remotely by a dementia treatment team through weekly Skype videoconferencing calls. Another team is currently conducting a trial (Malmgren Fänge et al., 2017) to send alerts (SMS and/or phone call) to the caregiver of a person with dementia if something unusual happens at home. The surveillance kit includes home-leaving sensors, smoke and water leak detectors, bed detectors, and automatic lights that monitor the person’s behavior.

## Future Directions for the Management of BPSDs in the Patient’s Home: Personalized Medicine and Digital Biomarkers

Aside from technological products that directly provide therapy, such as the Food and Drug Administration–approved PARO biofeedback device, a “pet seal” robot that adjusts its responses based on patient behavior and has demonstrated clinical benefits in BPSDs (Mervin et al., 2018), digital technologies are positioned to play a central role in transforming our therapeutic approach through more effective monitoring. Several authors consider them as a possible impetus for the implementation of a more personalized medicine, focused on the person rather than the disease (Insel, 2017; Antman and Loscalzo, 2016; Hird et al., 2016). This model of care would promote continuously updated and individualized treatment (Hood and Flores, 2012; Antman and Loscalzo, 2016). A recent consensus recommends this personalized approach as well as intensive home care based on new technologies for patients with dementia and their caregivers (Samus et al., 2018). A potential benefit is the opportunity to treat earlier, when symptoms can still be treated with existing nonpharmacological and pharmacological therapeutic solutions and to reassess treatments in a timely manner over time. Anticipation is extremely valuable in the management of BPSDs. New terms illustrating this technological reality are emerging in medical research: “digital biomarker” (Kramer et al., 2017; Califf, 2018), “electronic biomarker” (Faurholt-Jepsen et al., 2015), or “digital phenotyping” (Insel, 2017) (**Box 1**). These sensitive and continuous measures may even reveal subtle intraindividual changes or modified variability over time and may constitute new early biomarkers of mental health (Kaye, 2008; Dodge et al., 2014). Thus, these new biomarkers could detect mild or early BPSDs and then could be used to implement prevention strategies.

Box 1 **Digital biomarker definition.**

Objective, quantifiable, physiological, and/or behavioral data that are collected and measured by means of digital devices such as embedded environmental sensors, portables, wearables, implantables, or digestibles, and which opens up opportunities for the remote collection and processing of ecologically valid, real-life, continuous, long-term, health-related data.

**Figure 1:**
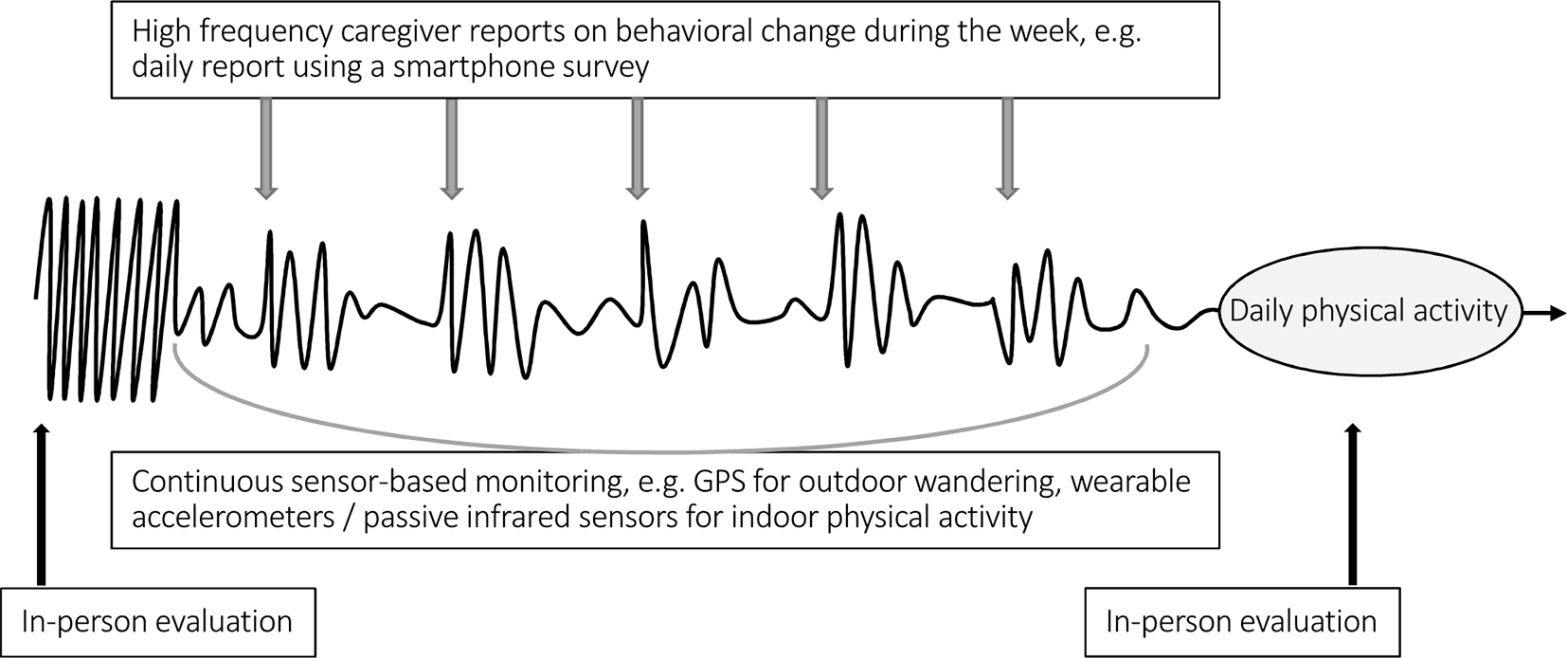
Example of a drug prescription sequence and complementarity of clinical and technology-based assessment. In this example, an increase in night wandering was the reason for prescribing a psychotropic drug (we are not discussing the relevance of the prescription). The curve represents physical activity over time. We observe the restoration of a day-night rhythm in the following days (spontaneous or consecutive to treatment’s effect). An increase in physical activity in the following nights or worrying inactivity during the day would, on the contrary, encourage a timely reassessment of the prescription. Information can be collected through caregiver input (e.g., semiautomated questionnaires on smartphones) and/or by sensors in addition to episodic clinical assessments.

While the inability to track changes in cognition, mood, and behavior over time is a major challenge in care, technological innovations suggest possible improvements in this area (Insel, 2017; Seelye et al., 2018). Coupling ICT terminals (e.g., touchpads) with wearable or embedded connected sensors could allow objective, high-frequency data collection in patients’ homes and would complement self-administered questionnaires (through an informant) and episodic clinical assessments (**Figure 1**). Advances in pervasive computing and high-dimensional data analysis have made this objective credible (Kaye et al., 2011; Lyons et al., 2015; Seelye et al., 2018). Several studies confirm the relationship between physiological parameters measured by sensors and cognitive, psychological, and behavioral outcomes. In a younger psychiatric population, data automatically generated using smartphones correlate with clinically rated symptoms in patients with bipolar disorder. According to Faurholt-Jepsen et al. (2015), such data could be used as an “electronic biomarker of illness activity.” Features extracted from GPS and mobile phone use also provided behavioral markers that were strongly linked to depressive symptoms (Saeb et al., 2015). In the field of cognitive impairment, more and more publications support the feasibility of long-term remote monitoring of cognition using innovative technologies (Piau et al., 2019). From a research perspective, many consider digital biomarkers as the path to a better understanding of disease processes and therefore to potentially groundbreaking research hypothesis. They could also contribute to more effective pharmaceutical research (**Box 2**, Dodge et al., 2015; Dorsey et al., 2015; Torous et al., 2015; Leurent and Ehlers, 2015; Teipel et al., 2018).

Box 2 **Information and Communication Technologies (ICT) and pharmaceutical research.**

ICT could contribute to more effective pharmaceutical research in several ways by

- targeting the earliest stage of behavioral change;
- reducing the sample size required (closer data points);
- providing more objective measures of behavior (outcome measurement);
- ensuring better monitoring of potential benefits and risks;
- ensuring better follow-up of cases of noncompliance;
- allowing a better understanding of intervention failures;
- providing a more in-depth understanding of disease processes;
- allowing the development of innovative pathophysiological hypotheses; and
- facilitating massive collection and processing of data (e.g., questionnaires).

From a much more concrete point of view and with regard to the choice of terminal, the desktop computer is the most widely proposed medium in the literature to communicate with family caregivers and in some cases collect sensor data. However, new interfaces seem more appropriate for home remote monitoring. Touchpads are commercially successful with the older population (Mobile fact sheet; Jenkins et al., 2016). Nevertheless, if we consider moving to a large-scale population-based evaluation, smartphones are the most mobile and ubiquitous device in the general population. They have the undeniable ability to reach a large population in a limited time (Dufau et al., 2011) and have also proven to be a feasible tool for the cognitive assessment of older people (Brouillette et al., 2013).

## Challenges and Illusions of New Technologies

Technological tools, like any part of medical intervention, carry potential limitations, risks, and ethical concerns. Measurements based on passive sensors and remote questionnaires, while complementary to “traditional” data collection techniques, have their own unresolved limitations (e.g., algorithm reproducibility in different contexts). In addition, and apart from the regulatory issues related to clinical trials, which are discussed in detail elsewhere (Hird et al., 2016), pervasive computing raises serious privacy and security issues. The challenge of health data security is far from being solved despite the development of international health data security standards (Health Insurance Portability and Accountability Act security compliance, Personal Data Protection Regulation of European Union). Another concern, which is not specific to technology, is the ethical issue of monitoring a person with cognitive impairment without his/her conscious consent at all time (e.g., GPS monitoring of elopement behavior). However, just as chemical restrictions had to be considered in the light of available alternatives, i.e., physical restrictions, the negative side effects of technologies must be considered in the overall context of suboptimal treatment of BPSDs.

Of equal concern is the widespread dissemination of commercial applications or devices aimed at improving the health of older people. Even if privacy issues are set aside, it is not yet clear whether these solutions could provide a direct or indirect benefit. In the field of cognitive impairment, the obstacles to developing effective tools should not be underestimated. Potentially relevant and “simple” ideas are struggling to meet initial expectations. One example is the use of electronic pill dispensers or smartphone reminder apps. While attractive for their simplicity, they have not been found to improve compliance in a sustainable way (Hird et al., 2016). Incentive solutions such as prompting involve complex technical installations to provide a contextualized reminder to the user. Reminding people to take a medication at an inappropriate time or place (e.g., in a car without water) will have a negative impact on the expected benefits. In addition, the incentive solutions ignore the conscious and voluntary aspect of nonadherence to drugs (Hird et al., 2016). Finally, while ICTs have the potential for real-time monitoring, most field studies have used retrospective data analysis, and this possibility has been relatively unexplored (Piau et al., 2019).

Another point to consider is the potential consequences of implementing these technologies in real life situations for the current health care organization (e.g., information overload). The first step in the large-scale clinical use of ICTs is to clarify their exact place and role in the clinical care pathway. Digital measurements require extensive data processing before they can be translated into clinical information relevant to health stakeholders. While we know why this monitoring is relevant, it is not yet clear who will receive the information and when, in what form, for what type of action, and, finally, who will pay for it. To date, the feasibility of integrating such a solution into complex and multidisciplinary clinical care networks is still unknown. Community-based studies can first be implemented on a large scale before the health care system is ready for change. However, replication in different settings will remain an important issue.

Finally, one of the major obstacles to the deployment of ICTs in the field is the technical literacy and acceptance of caregivers and therefore their ability to act as an intermediate “field worker” to provide information at a distance. The caregiver is often an older person who also has health problems. Nevertheless, given the growth in ICT adoption, it can be expected that this type of organization will be easier to generalize in the coming years (Mobile fact sheet) with the new generations to come. To overcome the limitations of technical literacy, it is also possible to consider only the basic functions of a smartphone (e.g., text messaging) or to involve a third party (e.g., home technical assistance). Another limitation is the acceptability of sensor-based measurements in a population living with cognitive impairment and anosognosia. The very low compliance rate (32%) of wearable activity trackers in a younger population (52 years old on average) evaluated in the very short term is anything but encouraging (van der Meij et al., 2018; Piau and Wild, 2019). The literature supports a better acceptability of embedded sensors for monitoring daily life, although they pose their own problems (e.g., difficulties in following two people in the same house) (Piau et al., 2019). If wearable sensors are still an option (Farina et al., 2019), it seems clear that, regardless of the ethical implications, total unobtrusiveness would be desirable (e.g., patch device).

## Conclusion

Behavioral and psychological symptoms of dementias challenge the traditional assessment of medical outcomes in clinical care and clinical trials. Self-evaluation is mostly unfeasible because of underlying dementia, and the evolving nature of the symptoms biases the heteroevaluation. Remote description at high frequency by caregivers and continuous monitoring by sensors can provide additional information. Most recent or ongoing scientific work on mental health and technologies supports digital biomarkers, not so much as diagnostic tools but rather as monitoring tools, an area where unmet needs are significant. Follow-up is (or should be) an integral part of therapy, especially in complex geriatric situations. However, interpreting sensors raw data is not straightforward. The measuring devices must be validated: we must ensure that the measurement is reliable and reproducible and that we interpret the results correctly. Information and communication technology–derived data could also improve BPSD knowledge and treatment procedures. Potentially innovative molecules could be tested in an environmentally friendly setting, and their effectiveness, as well as their side effects, could be characterized more easily and quickly.

## Author Contributions

AP, PR, and MM: drafting of the manuscript; FN: critical revision of the manuscript for important intellectual content.

## Conflict of Interest

The authors declare that the research was conducted in the absence of any commercial or financial relationships that could be construed as a potential conflict of interest.
